# Giant Anisotropic Magnetocaloric Effect in Double-perovskite Gd_2_CoMnO_6_ Single Crystals

**DOI:** 10.1038/s41598-017-16416-z

**Published:** 2017-11-23

**Authors:** J. Y. Moon, M. K. Kim, Y. J. Choi, N. Lee

**Affiliations:** Department of Physics and IPAP, Yonsei University, Seoul, 120-749 Korea

## Abstract

The magnetocaloric effect (MCE) is described by the change in temperature of a material by magnetic field variation and is a crucial subject in magnetism; it is motivated by the desire to enhance energy-efficient magnetic refrigeration for clean technology. Despite the recent discovery of the giant cryogenic MCE in double perovskites, the role of magnetic anisotropy has not yet been clearly discussed, because of the averaging effect of polycrystalline samples. Here, we investigated the anisotropic MCE in the single-crystal double perovskite Gd_2_CoMnO_6_. In addition to the ferromagnetic order of the Co^2+^ and Mn^4+^ moments, the large Gd^3+^ moments align below *T*_Gd_ = 21 K, exhibiting an isotropic nature. Because of the intricate temperature development of magnetically hysteretic behaviour and metamagnetism, the change in magnetic entropy along the *c*-axis appears to be relatively small. On the contrary, the smaller but almost reversible magnetization perpendicular to the *c-*axis leads to a large MCE with a maximum entropy change of 25.4 J/kg·K. The anisotropic MCE generates a giant rotational MCE, estimated as 16.6 J/kg·K. Our results demonstrate the importance of magnetic anisotropy for understanding the MCE and reveal essential clues for exploring suitable magnetic refrigerant compounds aiming at magnetic functional applications.

## Introduction

Magnetic materials exhibiting the giant magnetocaloric effect (MCE) have been widely investigated^1–3^, and it would be advantageous to replace conventional refrigeration based on vapor compression and realize energy-efficient magnetic refrigeration for clean technology. It is desirable to design and discover new compounds that exhibit the giant MCE for more feasible applications. The giant and/or reversible MCE near room temperature has recently been found in several alloy systems such as Gd_5_(Si_*x*_Ge_1−*x*_)^4^, MnFeP_0.45_As_0.55_^5^ (magnetic entropy change Δ*S*_*M*_ = 18.0 J/kg·K for Δ*H* = 0–5 T), Ni–Mn–In^6,7^ (adiabatic temperature change Δ*T*_*ad*_ = 6.2 K for Δ*H* = 0–1.9 T), and La(Fe,Si)_13_^8^ (Δ*S*_*M*_ = 16  kJ/m^3^·K for Δ*H* = 0–2 T), which offer potential refrigeration techniques for domestic usage and microelectronic devices. Cryogenic magnetic refrigeration is also essential for obtaining sub-Kelvin temperatures as a substitute for ^3^He/^4^He dilution refrigeration, whose cost continues to increase, and for hydrogen gas liquefaction, which is utilized as an alternative fuel. The MCE has been explored in many insulating oxides^9–13^, which can be easily manufactured on account of the chemical stability along with the avoidance of the refrigeration inefficiency driven by eddy current losses. Recently, the giant cryogenic MCE was discovered in several transition metal oxides such as Dy_2_CoMnO_6_^10^ (Δ*S*_*M*_ = 9.3 J/kg·K for Δ*H* = 0–7 T), HoMnO_3_^11^ (Δ*S*_*M*_ = 13.1 J/kg·K for Δ*H* = 0–7 T)., GdCrO_4_^12^ (Δ*S*_*M*_ = 29.0 J/kg·K for Δ*H* = 0–9 T), and HoCrO_4_^13^ (Δ*S*_*M*_ = 31.0 J/kg·K for Δ*H* = 0–8 T). However, most of the studies were performed on polycrystalline forms, preventing detailed characterization of the intrinsic properties of the giant MCE associated with magnetic and crystalline anisotropy.

**Figure 1:**
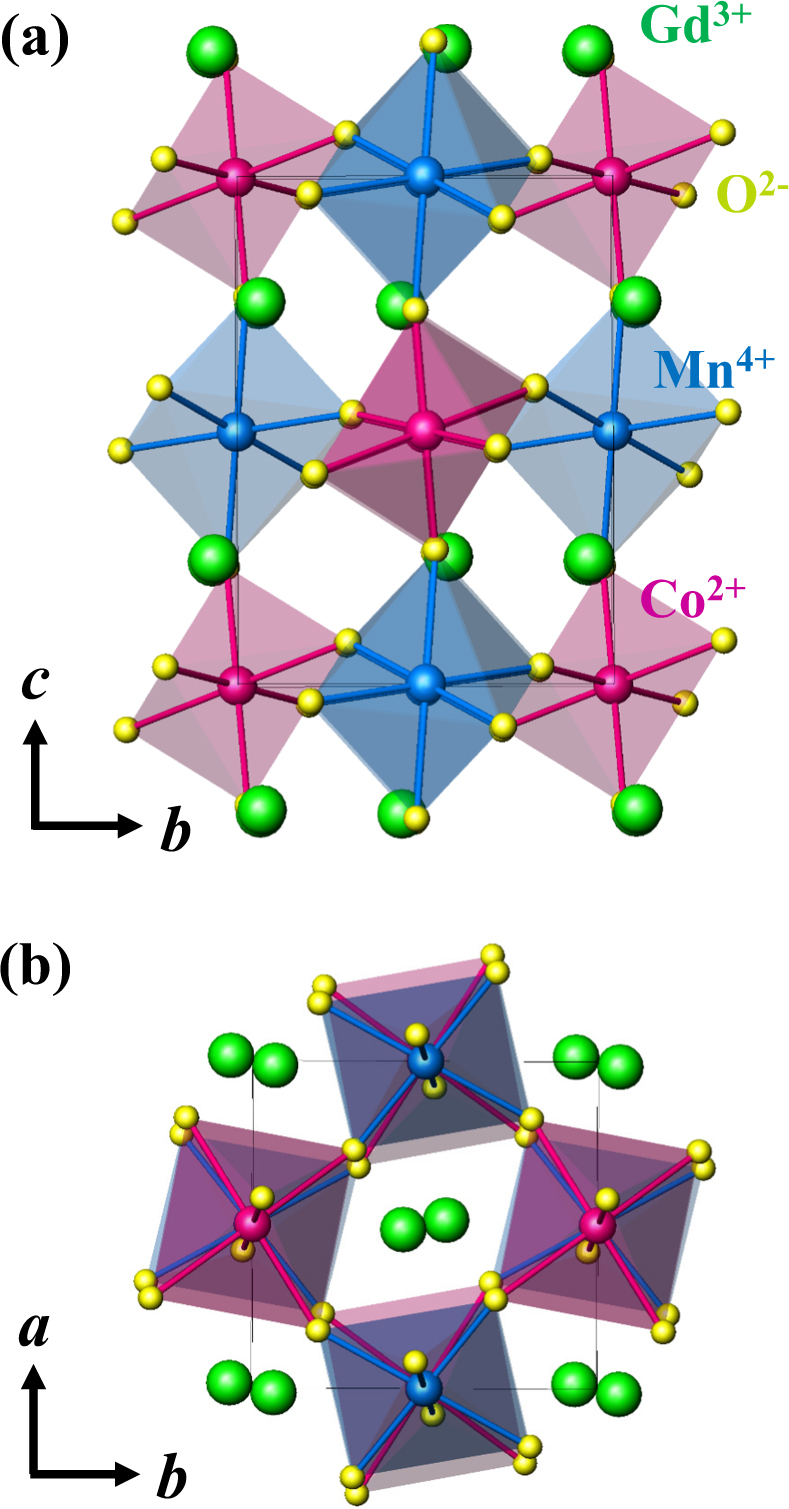
Crystallographic structure of a GCMO crystal. (**a**,**b**) Views of the crystal structure of double perovskite GCMO crystal from the *a-* and *c-*axes, respectively. The green, pink, blue, and yellow spheres represent Gd^3+^, Co^2+^, Mn^4+^, and O^2−^ ions, respectively.

To investigate the influence of the anisotropic characteristics on the giant MCE in one of the transition metal oxides, we have synthesized single crystals of the double perovskite Gd_2_CoMnO_6_ (GCMO) using the conventional flux method^14^. Double perovskite R_2_CoMnO_6_ (R = La, …, Lu) compounds, where Co^2+^ and Mn^4+^ ions are alternately located in corner-shared octahedral environments, exhibit assorted physical properties such as metamagnetism^15–17^, exchange bias^18,19^, the re-entrant spin-glass state^20,21^, and multiferroicity^16,22–24^ because of the intricate magnetic interactions and ionic valence/antisite disorders between mixed-valence magnetic ions. The ferromagnetic order originates from the dominant Co^2+^ and Mn^4+^ superexchange interactions, and its transition temperature varies linearly from 204 K for La_2_CoMnO_6_^25^ to 48 K for Lu_2_CoMnO_6_^26^ as the size of the rare earth ions decreases. GCMO crystallizes in a monoclinic *P2*_1_ */n* double-perovskite structure with a unit cell of *a* = 5.3158 Å, *b* = 5.6050 Å, *c* = 7.5759 Å, and *β* = 89.9541°. The crystal structures viewed from the *a-* and *c-*axes are depicted in Fig. 1(a) and (b), respectively. The oxygen octahedral cages are considerably distorted due to the comparatively small radius of the Gd^3+^ ion. In a previous study, the polycrystalline form of GCMO revealed a large maximum entropy change of Δ*S*_*M*_ *≈* 24 J/kg·K^27^, attributed to the large magnetic moments of Gd^3+^ ions.

From our examination of the anisotropic MCE in GCMO single crystals, we only found a Δ*S*_*M*_ value of half that in the polycrystalline specimen along the magnetic easy *c*-axis despite the large magnetization (*M*) at an applied magnetic field (*H*). The significant reduction in MCE was caused by the strong temperature (*T*) dependence of the magnetic hysteresis and metamagnetic transition. Instead, the isothermal *M* perpendicular to the *c-*axis exhibited almost reversible hysteretic behaviour, which contributed to the giant MCE associated with the magnetic entropy change Δ*S*_*M*_ = 25.4 J/kg·K and adiabatic temperature change Δ*T*_*ad*_ = 7.3 K in Δ*H* = 0–9 T. As a result, the highly-anisotropic Δ*S*_*M*_ produced a giant rotational MCE, estimated as 16.6 J/kg·K at 4 K. These results clearly suggest that a meticulous understanding of strongly anisotropic characteristics is crucial for finding improved functional properties in double-perovskite compounds.

## Results and Discussion

**Figure 2:**
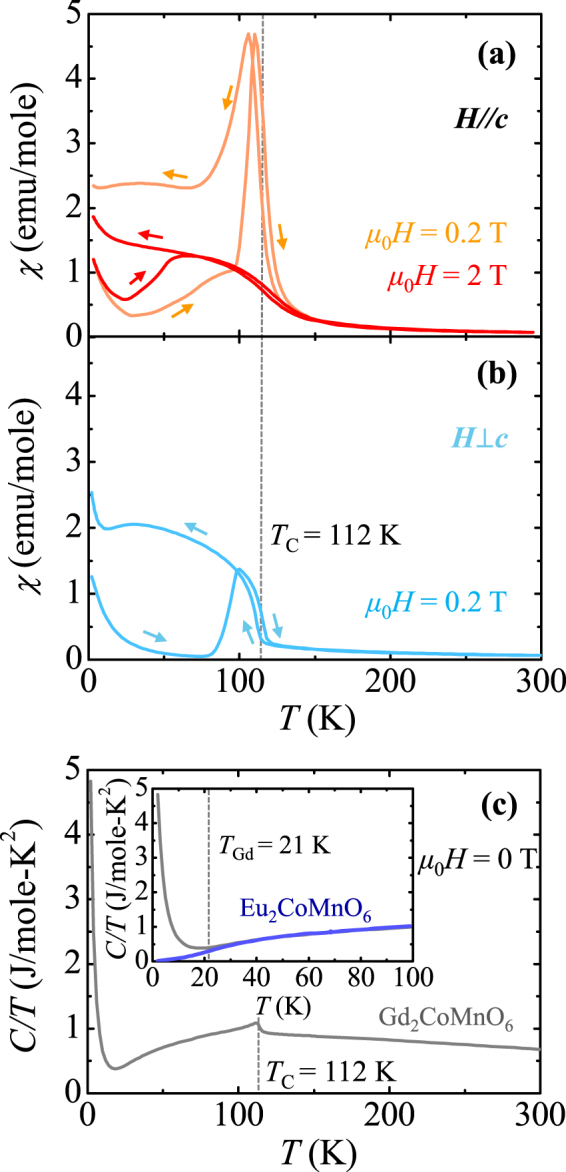
Characterization of temperature-dependent physical properties in a GCMO crystal. (**a**,**b**) Temperature dependence of the magnetic susceptibility, *χ* = *M/H*, of double perovskite GCMO single crystal parallel (*H*//*c*, *μ*_0_ *H* = 0.2 T and 2 T) and perpendicular (*H*⊥*c*, *μ*_0_ *H* = 0.2 T) to the *c*-axis, respectively, measured upon warming from 2 to 300 K after zero-field-cooling and upon cooling at the same field. The vertical dashed line indicates the ferromagnetic transition temperature, *T*_C_ = 112 K. (**c**) Temperature dependence of specific heat divided by temperature, *C/T*, measured at zero magnetic field. The inset shows a comparison of *C/T* up to 100 K between GCMO and Eu_2_CoMnO_6_ measured at zero magnetic field. The vertical dashed line denotes the ordering temperature of Gd^3+^ moments as *T*_Gd_ = 21 K.

The anisotropic magnetic properties of GCMO single crystals were examined parallel (*H*//*c*) and perpendicular to the *c-*axis (*H*⊥*c*). The *T* dependence of the magnetic susceptibility, *χ* = *M*/*H*, was measured upon warming at *μ*_0_ *H* = 0.2 T after zero-*H*-cooling (ZFC *χ*) and upon cooling at the same field (FC *χ*), as shown in Fig. 2(a) and (b), respectively. As *T* decreases, *χ* increases smoothly until exhibiting a sharp rise at *T*_C_ = 112 K, ascribed to the ferromagnetic order of the Co^2+^ (*S* = 3/2) and Mn^4+^ (*S* = 3/2) moments. The ferromagnetic behaviour was characterized by the positive Curie *T* determined by the Curie-Weiss law. *T*_C_ was determined by the *T* derivative of *χ* and by the sharp anomaly in the *T* dependence curve of the heat capacity divided by the temperature (*C/T*) at zero magnetic field (Fig. 2(c)). Reducing *T*_C_ further, FC *χ* reaches an approximate plateau. In contrast, ZFC *χ* decreases at the beginning of the warming from 2 K and the slope of *χ* changes at around *T*_Gd_ = 21 K, below which *C/T* exhibits an abrupt increase, indicating the ordering of the Gd^3+^ moments. Above *T*_Gd_, the *χ* increases gradually due to the thermally activated domain wall motions. Just below *T*_C_, the ZFC *χ* shows a distinct peak, which signifies an additional domain wall de-pinning process. At *μ*_0_ *H* = 2 T, ZFC and FC *χ*’s exhibit conventional ferromagnetic behaviour without any sharp anomaly near *T*_C_. The *T* at which the ZFC *χ* and FC *χ* curves start to split is observed, indicating the onset of magnetic irreversibility. The thermally hysteretic behaviour of the *χ* around *T*_C_ indicates the first-order nature of the transition. The *χ* for the two different orientations at *μ*_0_ *H* = 0.2 T exhibiting a strong magnetic anisotropy near *T*_C_ indicates that the Co^2+^ and Mn^4+^ spins are mainly aligned along the *c*-axis.

To estimate the entropy change based solely on the spin order of Gd^3+^ ions, Δ*S*_*Gd*_, the *C/T* for Eu_2_CoMnO_6_, which includes nonmagnetic Eu^3+^ ions with a similar ionic radius to Gd^3+^ ions, was measured, as shown in the inset of Fig. 2(c). The Δ*S*_*Gd*_ below *T*_Gd_ was obtained by integrating *C/T* by *T* (2–21 K) for GCMO after subtracting the data from Eu_2_CoMnO_6_. The calculated Δ*S*_*Gd*_ was 17.3 J/mole·K, which is 50% of the expected value of the fully saturated Gd^3+^ moments, i.e., 2*R* ln(2*J*+1) = 34.6 J/mole·K, where *R* is the gas constant and *J* is the total angular momentum (*J* = 7/2 for the Gd^3+^ ion).

**Figure 3:**
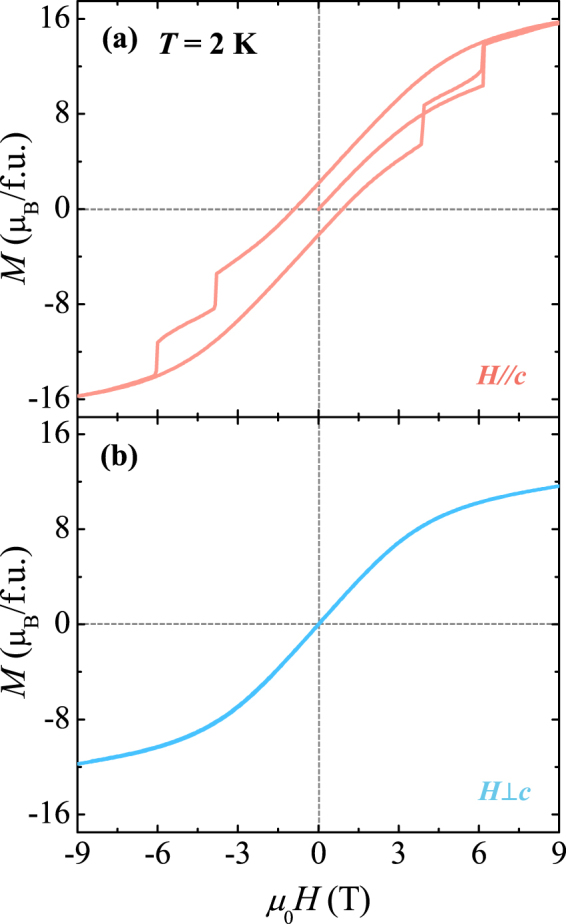
Anisotropic isothermal magnetization of a GCMO crystal at 2 K. (**a**) Full magnetic hysteresis curve of isothermal magnetization along the *c-*axis measured at 2 K up to *H* = 9 T. (**b**) Magnetic field dependence of magnetization perpendicular to the *c-*axis measured at 2 K up to *H* = 9 T.

Figure 3(a) and (b) display the isothermal *M* for the two different orientations, measured up to *μ*_0_ *H* = 9 T at 2 K. The initial *M* curve at *H*//*c* exhibits a gradual increase as *H* increases before a sudden jump at 6.2 T. The *M* at the maximum *H* of 9 T is found to be 15.7 *μ*_B_/f.u., which is 72% of the completely saturated moments by considering the effective magnetic moment of a Gd^3+^ ion as *μ*_Gd_ = 7.98 *μ*_B_. The consecutive sweeping of *H* between +9 and −9 T leads to the sharp double-step metamagnetic transitions at *H* = ±3.9 and ±6.2 T. Consequently, the full curve exhibits narrow hysteretic behaviour with the remanent *M* as *M*_*r*_ = 2.1 *μ*_B_/f.u. and the coercive field as *H*_*c*_ = 0.9 T. In contrast, the *M* at *H*⊥*c* varies smoothly without any magnetic hysteresis. Regardless of the hard magnetic axis for the ferromagnetic Co^2+^ and Mn^4+^ sublattice, the large magnetic moment of 11.6 *μ*_B_/f.u. at 9 T implies the somewhat isotropic nature of the Gd^3+^ spins associated with the half-filled 4 *f* electronic configuration. In other words, the difference of the *M* values at 9 T between the two orientations is caused by the Co^2+^ and Mn^4+^ spins mainly aligned along the *c*-axis.

The plausible cause for the features of the metamagnetic transitions^18,28^ found in the fully hysteretic *M* curve at *H*//*c* (Fig. 3(a)) can be determined from the distinctive magnetic anisotropy between Gd^3+^ and Co^2+^/Mn^4+^ moments. After ZFC, the Co^2+^ and Mn^4+^ moments are mostly in a parallel or antiparallel arrangement along the *c*-axis, while the Gd^3+^ moments are oriented in random directions reflecting the isotropic character. Upon increasing *H*, the continuous increase of *M* up to 10.4 *μ*_B_/f.u. is mainly caused by the alignment of Gd^3+^ moments along with the flipping of only the partial Co^2+^ and Mn^4+^ spins due to the large magnetic anisotropic energy. At 6.2 T, the abrupt jump of *M* occurs because the Zeeman energy of the Co^2+^/Mn^4+^ sublattice overcomes the anisotropic energy. The gradual decrease in *H* from +9 T indicates the reduction of *M* until it encounters the two consecutive metamagnetic transitions, originating from the flipping of the Gd^3+^ spins and then the Co^2+^ and Mn^4+^ spins, respectively. This assumption is compatible with the magnetically anisotropic energy of the Co^2+^ and Mn^4+^ spins being larger than that of the Gd^3+^ spins. Although the postulation of rather isotropic nature of Gd^3+^ moments gives moderate interpretation for isothermal *M* at 2 K, the narrow hysteretic behaviour with a small *M*_*r*_ may indicate small degree of interaction between Gd^3+^ and ferromagnetic Co^2+^/Mn^4+^ sublattices. Upon decreasing *H* from +9 T, the negative exchange coupling between Gd^3+^ and Co^2+^/Mn^4+^ spins accompanied by a smaller magnetocrystalline anisotropy energy and larger moment of Gd^3+^ ions leads to the progressive decrease in the net Gd^3+^ moments, followed by the considerable reduction of *M*_r_.

**Figure 4:**
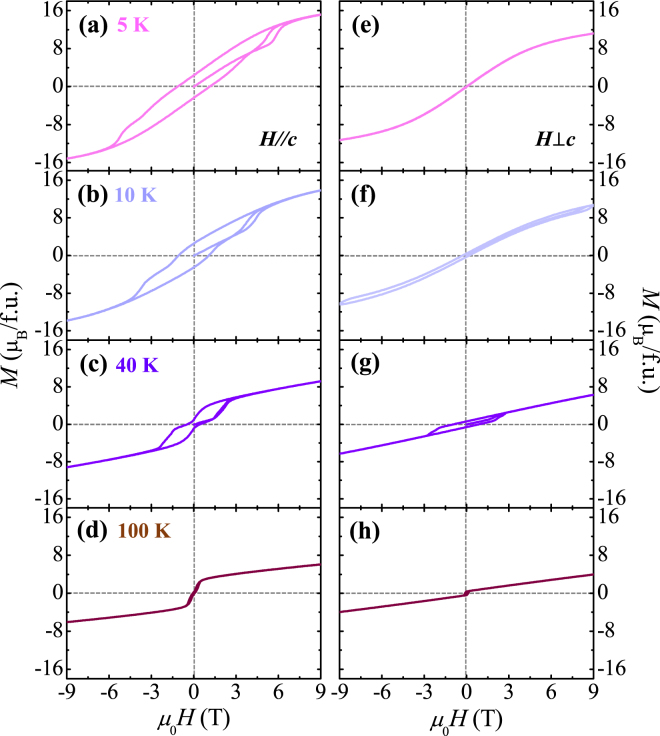
Temperature development of isothermal magnetization. Isothermal magnetizations (**a**–**d**) parallel and (**e**–**h**) perpendicular to the *c-*axis measured at *T* = 5, 10, 40 and 100 K, respectively, up to *H* = 9 T.

Figure 4 presents the full anisotropic curves of *M* up to 9 T at various *T* (*T* = 5, 10, 40, and 100 K). At 5 K and *H*//*c*, the double-step metamagnetic transitions broaden, while the remanent *M* and coercive field appear to be enhanced as *M*_*r*_ = 2.4 *μ*_B_/f.u. and *H*_*c*_ = 1.1 T. At 10 K, the area inside the magnetic hysteresis loop along *H*//*c* is considerably reduced with the shift in the metamagnetic transitions to lower *H*, but *M*_*r*_ and *H*_*c*_ are almost maintained. At *H*⊥*c*, a very narrow but large magnetic hysteresis loop is observed, presumably because of a weakened magnetic anisotropy due to thermal energy. As *T* increases further, the area of the magnetic hysteresis loop rapidly shrinks and *M*_*r*_ and *H*_*c*_ also decrease. At 100 K, a slight hysteretic behaviour remains but the metamagnetic transitions almost vanish.

**Figure 5:**
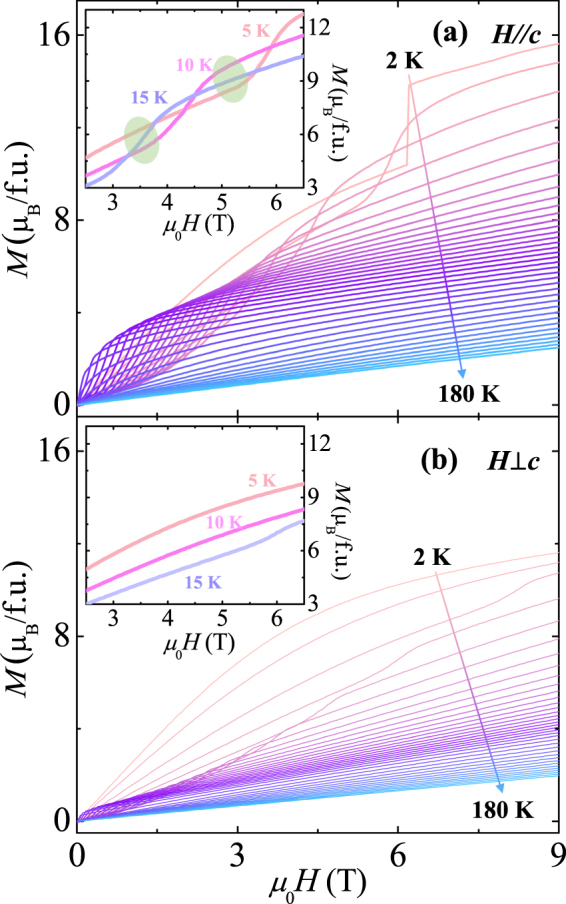
Initial magnetization curves in a wide range of temperatures. (**a**) Initial curves of isothermal magnetization at *H*//*c* and various temperatures varying from 2 to 180 K. The inset shows the magnified region of magnetization for *T* = 5, 10, and 15 K. The green shaded ellipses indicate the reversed order of magnetization magnitudes due to the shift in metamagnetic transitions according to *T*. (**b**) Initial curves of isothermal magnetization at *H*⊥*c* and various temperatures varying from 2 to 180 K. The inset shows the magnified region of magnetization for *T* = 5, 10, and 15 K.

Based on the distinctive magnetic properties for the two different orientations, an anisotropic MCE in the GCMO was obtained by measuring the initial *M* curves with dense *T* steps ranging from 2 to 180 K in Fig. 5. In contrast to the typical reduction of *M* values with the increase in *T*, the initial *M* curves at *H*//*c* develops in a complicated manner. The sharp steps of the metamagnetic transitions at 2 K move progressively to lower *H* and become broader as *T* increases. For this reason, the *M* value in a given *H* regime is lower than that at higher *T*. In the inset of Fig. 5(a), the isothermal *M* values measured at 5, 10, and 15 K are magnified. The green shaded areas represent specific examples of the reversed order of magnitude for the *M* values. As *T* is further increased, the occurrence of the reversed order shifts gradually to the lower *H* regime. At *H*⊥*c*, a small but broad transition feature also occurs at some temperature regime while moving to lower *H* as *T* is further increased, however, the overall magnitude of *M* is reduced in most of the regime of *H* with increasing *T*, as shown in the inset of Fig. 5(b).

**Figure 6:**
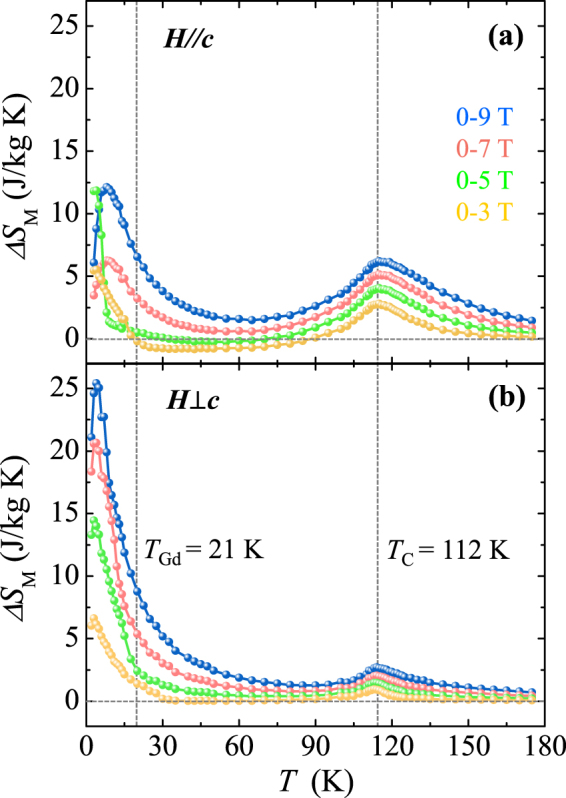
Anisotropic magnetocaloric effect in GCMO. Temperature dependence of magnetic entropy change, Δ*S*_*M*_, obtained by integrating the temperature gradient of the initial magnetization curves in Fig. 4 for (**a**) *H*//*c* and (**b**) *H*⊥*c* with magnetic field regimes of Δ*H* = 0–3 T (yellow), 0–5 T (green), 0–7 T (red), and 0–9 T (blue).

At a given *T*, the isothermal magnetic entropy change, Δ*S*_*M*_, can be obtained from the initial *M* curves using the Maxwell relation:

1 $${\rm{\Delta }}{S}_{M}(T,H)=-{\mu }_{0}{\int }_{0}^{{H}_{f}}\frac{\partial M(T,H)}{\partial T}dH\,$$

where *μ*_0_ is the magnetic permeability in vacuum, *H*_*f*_ is the end point of *H* for the integral (*H*_*f*_ = 3, 5, 7, and 9 T), and the *T* gradient of *M*, $\frac{\partial M(T,H)}{\partial T}$, was calculated approximately from the slope of two adjacent data points. The *T* dependence of the estimated entropy changes, Δ*S*_*M*_(*T*), are plotted in Fig. 6(a) and (b), respectively for *H*//*c* and *H* ⊥*c* with the *H* regimes of Δ*H* = 0–3, 0–5, 0–7, and 0–9 T. The Δ*S*_*M*_ values for both orientations exhibit broad peaks at *T*_C_, where the Δ*S*_*M*_ values are found to be 6.2 and 2.7 J/kg·K for *H*//*c* and *H* ⊥*c*, respectively. The much larger magnitude of Δ*S*_*M*_ at *T*_C_ for *H*//*c* describes the magnetic easy *c-*axis with respect to the ferromagnetic order of Co^2+^ and Mn^4+^ moments. At *H*//*c*, even with the large *M* at 2 K, the intercrossed isothermal *M* values due to the *T* development of metamagnetic transitions, as depicted in Fig. 5(a), results in a substantial cancellation of Δ*S*_*M*_. Consequently, a complicated *T* dependence of Δ*S*_*M*_ is observed below *T*_Gd_ for Δ*H* = 0–3 and 0–5 T, and even negative values of Δ*S*_*M*_ are revealed for *T* = 20–90 K. The maximum Δ*S*_*M*_ of 12.1 J/kg·K, found at 8 K for Δ*H* = 0–9 T, is smaller than the magnitude from the recent observation in the polycrystalline specimen. At *H* ⊥*c*, the near-absence of the estimated loss for Δ*S*_*M*_ generates a large magnitude of Δ*S*_*M*_, shown as a peak at low *T*, followed by a steep decrease in Δ*S*_*M*_. The maximum MCE at 5 K for Δ*H* = 0–9 T is estimated as Δ*S*_*M*_ = 25.4 J/kg·K.

**Figure 7:**
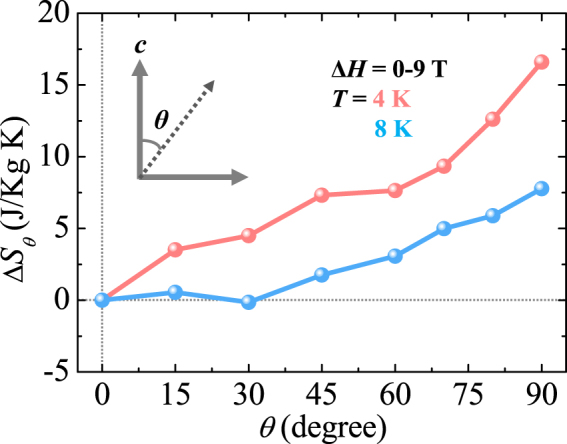
Rotating magnetocaloric effect in GCMO. Angular dependence of magnetic entropy change, Δ*S*_*θ*_, at *T* = 4 and 8 K with Δ*H* = 0–9 T. *θ* is the angle deviating from the *c*-axis, i.e., *θ* = 0° for *H*//*c* and 90° for *H* ⊥*c*.

By taking advantage of the strong magnetic anisotropy due to the distinctive characteristics of the double perovskite GCMO compound, the rotating MCE was measured by the angular dependence of Δ*S*_*M*_, denoted as Δ*S*_*θ*_, where *θ* is the angle deviating from the *c*-axis, i.e., *θ* = 0° for *H*//*c* and *θ* = 90° for *H* ⊥*c* (Inset of Fig. 7). Figure 7 shows Δ*S*_*θ*_ obtained at 4 and 8 K for Δ*H* = 0–9 T. As there is a different *T* dependence of Δ*S*_*M*_ between *H*//*c* and *H* ⊥*c*, the angle-dependent modulation of Δ*S*_*θ*_ varies strongly with *T*. At 8 K, Δ*S*_*θ*_ negligibly changes with the rotation of *θ* to 30° and increases linearly above 30°. The maximum Δ*S*_*θ*_ was evaluated as only 7.8 J/kg·K. At 4 K, on the contrary, the continued variation of Δ*S*_*θ*_ by *θ* rotation generates a giant rotational MCE as the maximum change of 16.6 J/kg·K, which would be beneficial for rotary magnetic refrigerator technology. The maximum difference of Δ*S*_*θ*_ in the GCMO is comparable to the other rotating magnetic refrigerants such as HoMn_2_O_5_^29^ (12.4 J/kg·K for Δ*H* = 0–7 T) and DyNiSi^30^ (17.6 J/kg·K for Δ*H* = 0–5 T).

**Figure 8:**
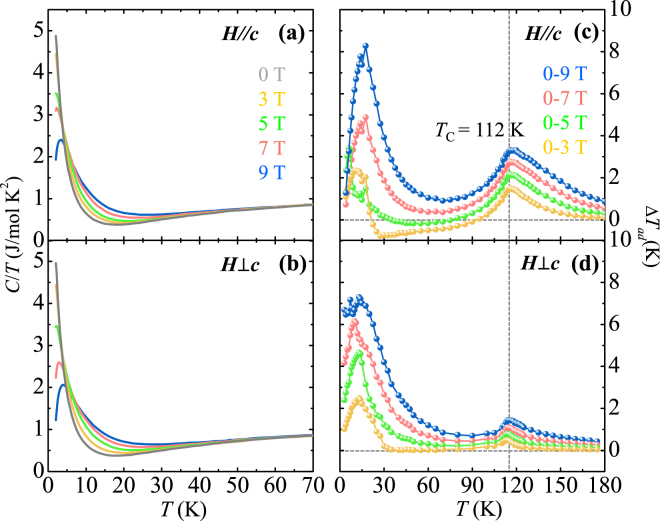
Adiabatic temperature changes in GCMO. (**a**,**b**) Temperature dependence of *C/T*, measured at (**a**) *H*//*c* and (**b**) *H*⊥*c* with various magnetic fields, *H* = 0, 3, 5, 7, and 9 T, shown at *T* = 2–70 K. (**c**,**d**) Temperature dependence of adiabatic temperature change, Δ*T*_*ad*_, estimated from integrating the temperature gradient of the initial magnetization curves multiplied by the corresponding reciprocal of *C/T* for (**c**) *H*//*c* and (**d**) *H*⊥*c* with magnetic field regimes of Δ*H* = 0–3 T (yellow), 0–5 T (green), 0–7 T (red), and 0–9 T (blue).

A more feasible aspect of MCE can be attained as the adiabatic *T* change, Δ*T*_*ad*_, from the following equation:

2 $${\rm{\Delta }}{T}_{ad}(T)=-{\mu }_{0}{\int }_{0}^{{H}_{f}}\frac{T}{C(T,H)}\frac{\partial M(T,H)}{\partial T}dH$$

where *C*(*T*, *H*) is the heat capacity at a given *T* and *H*. In many cases, *C* appears to be independent of the applied *H*, thus, it can be considered as a constant for the integral. However, in GCMO, the *T* dependence of *C/T* clearly varies depending on the magnitude of the applied *H*, as shown in Fig. 8(a) and (b) for *H*//*c* and *H* ⊥*c*, respectively, measured at *H* = 0, 3, 5, 7, and 9 T. At *H*//*c*, the *C/T* at very low temperatures decreases with *H* along with the emergence of a broad peak shifting to higher *T*. As *T* increases further, *C/T* decreases more slowly as *H* gradually increases. Therefore, the order of magnitude of *C/T* with respect to *H* is reversed at about 4 K. The *C/T* for *H* ⊥*c* exhibits similar *T* and *H* dependences as *H*//*c*. The *C/T* exhibits a greater reduction as *H* increases at the very low *T* regime with a further shift of the peak to higher *T*. Δ*S*_Gd_ is also estimated from *C/T* taken at 9 T for *H*//*c*. Δ*S*_Gd_ = 23.7 J/mole·K, which is about 69% of the value, assuming full saturation of Gd^3+^ moments, consistent with the measured magnetization at 9 T (Fig. 3(a)). Figure 8(c) and (d) display the *T* dependence of Δ*T*_*ad*_, estimated for *H*//*c* and *H* ⊥*c*, respectively, with Δ*H* = 0–3, 0–5, 0–7, and 0–9 T. At *H*//*c*, the *T* dependence of Δ*T*_*ad*_ behaves similarly to that of Δ*S*_*M*_. For Δ*H* = 0–9 T, starting from Δ*T*_*ad*_ = 1.3 K at 2 K, Δ*T*_*ad*_ increases with *T* and reaches 8.3 K at the peak position as *T* = 17.0 K. However, the estimated *T* dependence of Δ*T*_*ad*_ for *H* ⊥*c* was strongly influenced by the reciprocal of *C/T* during the calculation of the integral. Interestingly, the Δ*T*_*ad*_ for Δ*H* = 0–9 T maintains its magnitude between 6.5 and 7.3 K up to *T* = 17.0 K, suggesting that the MCE steadily covers the wide range of the low *T* regime for *H* ⊥*c*.

In summary, we explored the anisotropy of the magnetic and magnetocaloric properties of single-crystal double perovskite GCMO. Contrary to the anticipated large MCE along the magnetic easy *c*-axis, we attained a maximum entropy change of only half the magnitude of that found in the polycrystalline specimen. This substantial reduction is attributed to the intricate temperature evolution of metamagnetic transitions. Alternatively, an almost reversible hysteretic behaviour of isothermal magnetization perpendicular to the *c*-axis results in a large entropy change of Δ*S*_*M*_ = 25.4 J/kg·K, and thus the giant rotational MCE is taken as Δ*S*_*θ*_ = 16.6 J/kg·K at 4 K. The strongly anisotropic magnetic properties of the double-perovskite compound offer essential clues for the fundamental and applied research on magnetic materials, aiming to enhance the functional properties.

## Methods

Rod-shaped single crystals of GCMO were grown using the conventional flux method with Bi_2_O_3_ flux in air. The stoichiometric ratio of Gd_2_O_3_, Co_3_O_4_, and MnO_2_ powders was mixed and ground in a mortar, followed by pelletizing and calcining at 1000 °C for 12 h. The calcined pellet was reground and sintered at 1100 °C for 24 h. The same sintering procedure after regrinding was performed at 1200 °C for 48 h. A mixture of pre-sintered polycrystalline powder and Bi_2_O_3_ flux at a 1:12 ratio was heated to 1300 °C in a Pt crucible. It was melted at the soaking temperature for 5 h, slowly cooled to 985 °C at a rate of 2 °C/h, and cooled to room temperature at a rate of 250 °C/h. The temperature and magnetic field dependences of the DC magnetization, *M*, were examined by a vibrating sample magnetometer at *T* = 2–300 K and *H* = −9-9 T using a Physical Properties Measurement System (PPMS, Quantum Design, Inc.). The temperature dependence of specific heat, *C*, at various magnetic fields was measured with the standard relaxation method using the PPMS.
